# Propensity Score-Matched Comparison of Six-Strand All-Inside and Conventional Four-Strand Hamstring Autografts for ACL Reconstruction

**DOI:** 10.3390/jcm14176010

**Published:** 2025-08-25

**Authors:** Young Jin Seo, Si Young Song, Dongju Kim

**Affiliations:** Department of Orthopedic Surgery, Dongtan Sacred Heart Hospital, Hallym University College of Medicine, 7, Keunjaebong-gil, Hwaseong-si 18450, Republic of Koreadkmn123@naver.com (D.K.)

**Keywords:** anterior cruciate ligament, all-inside, reconstruction, hamstring, autograft

## Abstract

**Background/Objectives:** All-inside ACL reconstruction has emerged as a minimally invasive alternative to conventional techniques, with potential advantages in graft configuration and reduced surgical trauma. This study aimed to compare the clinical outcomes of all-inside and full tibial tunnel ACL reconstruction, focusing on graft diameter, postoperative stability, and functional recovery. We hypothesized that the all-inside technique would allow for thicker grafts and yield superior postoperative knee stability and functional outcomes, with postoperative anterior laxity as a major outcome of interest. **Methods:** This retrospective comparative study reviewed patients who underwent ACL reconstruction between January 2020 and February 2024. From January 2020 to September 2022, a four-strand hamstring autograft with full tibial tunnel technique (FT-4) was used, while from September 2022, a six-strand hamstring autograft with the all-inside technique (AI-6) was adopted to enable thicker grafts and optimize fixation. Among a total of 103 patients, 1:1 propensity score matching (PSM) was performed based on age, sex, BMI, laterality, ALL reconstruction, meniscal lesion, and preoperative anterior laxity (SSD). Graft diameter and clinical outcomes, including knee stability and functional scores, were compared between the matched groups. **Results:** After PSM, two comparable groups of 29 patients each were established. Graft diameter was significantly larger in the AI-6 group (9.5 ± 0.7 mm) compared to the FT-4 group (7.8 ± 0.8 mm, *p* < 0.001), while other baseline characteristics remained well balanced between the groups. At the final follow-up, both groups exhibited significant improvements in anterior laxity, functional scores, and pivot shift grades (all *p* < 0.001). The AI-6 group demonstrated superior outcomes with a significantly higher Lysholm score (82.2 ± 6.7 vs. 75.6 ± 8.9, *p* = 0.002), lower WOMAC score (8.0 ± 4.3 vs. 12.9 ± 10.5, *p* = 0.023), and reduced anterior laxity (1.6 ± 1.1 mm vs. 2.5 ± 1.4 mm, *p* = 0.005) compared to the FT-4 group, whereas no significant differences were observed in the IKDC, Tegner, Korean knee score, or pivot shift test results. A simple linear regression revealed a significant negative correlation between graft diameter and postoperative anterior laxity (B = −0.398, *p* = 0.048). **Conclusions:** The present study demonstrated that the use of a six-strand hamstring graft configuration in the AI-6 technique resulted in significantly thicker grafts and was associated with reduced postoperative anterior knee laxity compared to the FT-4 technique. While interpretation of these findings requires caution in light of MCID thresholds, the AI-6 group showed favorable outcomes in anterior laxity and selected functional scores, such as the Lysholm and WOMAC. This technique may offer practical clinical value, particularly in populations prone to smaller graft diameters, as it facilitates adequate graft thickness through multifold preparation, with the all-inside approach accommodating the inherent graft shortening.

## 1. Introduction

The conventional approaches for anatomical anterior cruciate ligament (ACL) reconstruction, including the modified transtibial, anteromedial portal, and outside-in techniques, have been widely accepted. Meanwhile, all-inside ACL reconstruction has emerged as an alternative technique characterized by tibial socket preparation and the potential for combination with various femoral tunnel methods, with the aim of improving clinical outcomes. Originally facilitated by the development of retrograde drilling systems in the early 2000s, the technique was later refined and popularized by Lubowitz, who emphasized its minimally invasive nature and the advantages of controlled intra-articular fixation [1,2,3,4]. The key differences between these approaches lie in the tibial tunnel drilling technique, graft preparation, and fixation strategies. These technical differences have sparked discussions regarding their potential impact on biomechanical and functional outcomes [5,6,7,8].

Traditional anatomical ACL reconstruction employs a full-length tibial tunnel, where a consistent diameter tunnel is drilled from the tibial cortex to the intra-articular exit. In contrast, all-inside ACL reconstruction utilizes a socket-based tunnel design, where only a short socket is created at the tibial insertion sites, precisely matching the diameter and length of the graft [9].

This technique features a distinct tibial-side fixation method compared to the standard approach. In traditional ACL reconstruction with the hamstring autograft, the graft that extends outside the tibial tunnel is additionally secured using a screw and washer or a spike. In contrast, the all-inside technique employs an adjustable loop cortical button on both the femoral and tibial sides, allowing for controlled graft tensioning within the sockets and minimizing metal irritation on the tibial side [9].

Another distinction between the two techniques is the preparation of the hamstring autograft. In traditional ACL reconstruction, the hamstring tendons are typically folded twice to create a sufficiently strong graft. However, in cases where the harvested tendons are relatively small or when only the semitendinosus is used, this configuration may result in a smaller graft diameter, which could potentially increase the risk of graft failure in high-demand athletes or individuals with larger physiques [10,11,12,13,14]. 

In contrast, 5- or six-strand graft configurations form thicker grafts and offer a reliable method to increase the diameter of relatively small hamstring autografts, helping to avoid failures related to undersized grafts [15]. The ability to prepare a thicker graft, which results in a shorter graft length due to increased folding, represents a potential advantage of the all-inside technique over conventional ACL reconstruction, as the shorter graft is well suited for socket-based tunnel preparation [16]. A recent meta-analysis reported that all-inside ACL reconstruction was superior to full tibial tunnel ACL reconstruction in terms of functional outcomes and tibial tunnel expansion, while postoperative anterior translation was comparable between the two techniques [3]. However, in most of the cited studies, a four-strand semitendinosus graft was used for the all-inside group, whereas the full tibial tunnel group utilized doubled gracilis and semitendinosus tendons. This differs from the present study, in which a six-strand hamstring autograft was used in the all-inside group, potentially influencing graft diameter outcomes.

Therefore, this study aims to compare the clinical effectiveness of all-inside ACL reconstruction with traditional anatomical ACL reconstruction, focusing on key outcome measures such as graft thickness, postoperative stability, functional recovery, and patient satisfaction. We hypothesize that ACL reconstruction utilizing the all-inside technique in combination with a six-strand hamstring autograft will yield a greater graft diameter, which in turn will be associated with reduced postoperative knee laxity and improved functional outcomes compared to the conventional anatomical reconstruction technique employing a four-strand configuration.

## 2. Materials and Methods

### 2.1. Demographics

This study was approved by our Institutional Review Board and Ethics Committee (File No. HDT 2025-01-009-001). A retrospective review was conducted on consecutive patients who underwent anterior cruciate ligament reconstruction between January 2020 and February 2024. During the initial period from January 2020 to September 2022, ACL reconstruction was performed using a 4-strand hamstring autograft with the standard full tibia technique (FT-4). From September 2022 to February 2024, the surgical technique was modified to a 6-strand hamstring autograft utilizing the all-inside approach (AI-6). This transition was made to accommodate a thicker graft configuration, which may offer greater mechanical strength and the potential for improved long-term graft integrity, while also optimizing fixation and minimizing bone loss.

Patients were included if they were between 18 and 49 years of age and had undergone primary ACL reconstruction using either the FT-4 or AI-6 technique. Only those with complete ACL tears, confirmed through clinical examination and MRI, and with a minimum follow-up of 12 months were eligible for the study. Patients were excluded if they had multi-ligament knee injuries requiring additional reconstruction, underwent revision ACL surgery, or had associated fractures or osteochondral injuries necessitating additional procedures. Other exclusion criteria included the presence of infection, inflammatory arthritis, or an incomplete follow-up. All the included cases were unilateral, and the analysis was therefore performed on a per-patient basis.

All surgeries were performed by a single experienced surgeon (YJS) using the respective technique based on the time period of intervention.

Clinical and functional outcomes were assessed preoperatively and at the last postoperative follow-up. Postoperative follow-up was conducted at 1 month, 3 months, 6 months, and 12 months, and subsequently at annual intervals.

Both groups followed the same standardized rehabilitation protocol. Postoperative rehabilitation included immediate range of motion exercises and partial weight-bearing with crutches as tolerated. Full weight-bearing was generally allowed by 4 weeks after surgery. Closed-chain exercises and proprioceptive training were initiated starting from 4 weeks postoperatively. Return to sports activities was typically permitted at 6 to 9 months, depending on individual progress.

Complications were defined as any postoperative adverse event requiring additional medical or surgical management beyond routine rehabilitation and follow-up care. Based on this definition, one patient in the FT-4 group developed a postoperative flexion contracture that required two arthroscopic adhesiolysis procedures. A residual contracture of 5° was observed at final follow-up. No other postoperative complications occurred in either group, and no perioperative complications were identified.

### 2.2. Surgical Technique

In both groups, the femoral tunnel was established using either the outside-in approach or the transportal technique, depending on intraoperative considerations. According to the senior surgeon’s operative strategy, the outside-in technique was favored in cases where preservation of the femoral remnant was feasible, as it provided better control over tunnel placement while minimizing disruption of the remaining native ACL fibers. In contrast, when the femoral side was characterized by an empty wall or minimal residual tissue, the transportal technique was employed.

In the outside-in technique, the femoral tunnel was created under visualization through the posteromedial portal. After inserting the arthroscope via the posteromedial portal, partial septal resection was performed to allow detailed visualization of the inner wall of the lateral femoral condyle, facilitating accurate tunnel placement. The femoral tunnel was positioned precisely at the anatomic ACL femoral footprint, targeting the anteromedial (AM) bundle’s direct fiber attachment site. Using a retrograde FlipCutter (Arthrex, Naples, FL, USA), the femoral socket was drilled approximately 7 mm shorter than the total measured length to prevent outer cortex breakage. Alternatively, the femoral tunnel was created using the transportal technique. Under direct visualization through the anteromedial (AM) portal, the femoral tunnel was drilled at the same position described earlier, with the knee hyperflexed to over 120 degrees. The reamer was introduced via the far anteromedial portal to achieve the desired tunnel placement.

**Figure 1:**
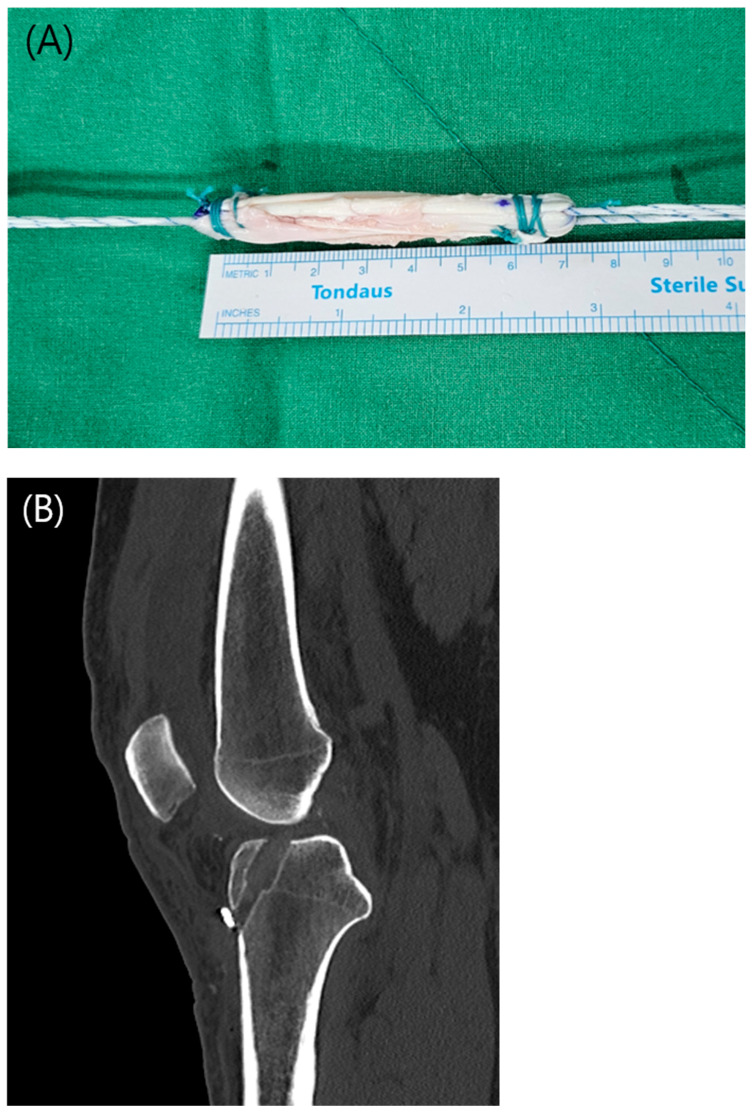
(**A**) Final construct of a 6-strand hamstring autograft. (**B**) Sagittal computed tomography image showing the configuration of the tibial tunnel after all-inside ACL reconstruction.

In the all-inside ACL reconstruction technique (AI-6 group), the graft was prepared using a triple-folded 6-strand hamstring autograft technique. Both the semitendinosus and gracilis tendons were harvested and whipstitched at both ends. Using a harvested hamstring length of approximately 22–25 cm, the final graft length was adjusted to approximately 7.5 cm (Figure 1(A)).

An adjustable cortical suspensory device was secured at each end of the folded graft to ensure proper fixation [17]. On the tibial side, the tibial socket was created in the AM bundle orientation at the native ACL footprint using a FlipCutter (Arthrex, Naples, FL, USA). To ensure that the combined length of the femoral tunnel, tibial tunnel, and intra-articular segment exceeded the total graft length, the angle of the tibial tunnel guide was adjusted accordingly (Figure 1(B)).

Before graft passage, leading sutures were advanced through the femoral and tibial tunnels and retrieved via the anteromedial portal. The sutures connected to the adjustable cortical suspensory devices at each graft end were then linked to the corresponding leading sutures. The graft was first passed and secured on the femoral side, followed by tibial passage and fixation, ensuring proper tensioning and stabilization.

**Figure 2:**
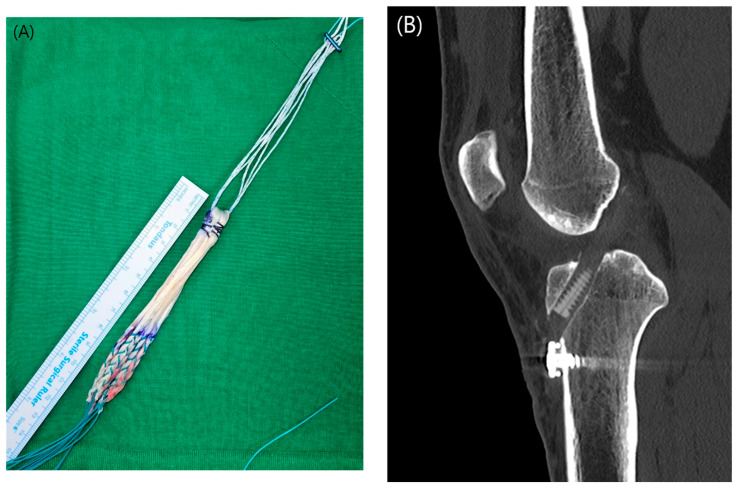
(**A**) Completed configuration of a four-strand hamstring autograft. (**B**) Sagittal computed tomography scan depicting the morphology of the full-length tibial tunnel after standard ACL reconstruction.

In the standard full tibia group (FT-4 group), a full-length tibial tunnel was drilled using the conventional technique, starting from the anteromedial cortex. The graft was prepared using a double-folded 4-strand hamstring autograft, utilizing both the semitendinosus and gracilis tendons (Figure 2(A)).

Fixation in the FT-4 group was achieved using an adjustable suspensory device on the femoral side. On the tibial side, an interference screw was used for primary fixation, with additional fixation performed using a screw and spike washer (Figure 2(B)).

For both the AI-6 and FT-4 groups, anterolateral ligament (ALL) reconstruction was performed in patients with a pivot shift grade 2 or higher, particularly those actively participating in sports. ALL reconstruction was performed using a 5 mm allogeneic tibialis tendon. The graft was fixed on the femoral side just posterior and proximal to the lateral femoral epicondyle using either a SwiveLock^®^ anchor (Arthrex, Naples, FL, USA) or a 6 mm interference screw. Tibial fixation was performed with a 6 mm interference screw at a point 15 mm below the joint line, midway between Gerdy’s tubercle and the fibular head, with the knee in 30° flexion and neutral rotation.

### 2.3. Outcome Measure

Graft diameter is recorded during surgery using a graft-sizing block (Arthrex, FL, USA) and defined as the largest diameter of the graft that could pass through the block. Meniscus injuries were documented based on their location (medial or lateral) and classified by type, including ramp lesions, root tears, longitudinal tears, horizontal tears, and others. Additionally, the treatment approach—whether repair or resection—was recorded for each case. The presence or absence of anterolateral ligament reconstruction was also documented.

Anterior laxity is assessed using the Telos device (Telos GmbH, Hennigsdorf, Germany), which quantifies the side-to-side difference (SSD) in anterior tibial translation under a standardized force of 150 Newtons. The pivot shift test is graded on a scale from 0 to 3, and was performed by an experienced senior surgeon (YJS). Functional knee scores, including the International Knee Documentation Committee (IKDC) Subjective Knee Evaluation Form, Lysholm, Tegner, and Western Ontario and McMaster Universities Osteoarthritis Index (WOMAC), Korean knee score (KKS), were recorded. All the assessments were conducted preoperatively and at the final follow-up during outpatient visits. The WOMAC and KKS were only assessed at the final follow-up.

The score assessments were performed by a single evaluator who was blinded to the patient information.

### 2.4. Statistical Analysis

All statistical analyses were performed using SPSS for Windows (version 29.0, SPSS Inc, Chicago, IL, USA). The raw data was organized in an Excel (Microsoft, version 16.0) sheet and uploaded to SPSS for analysis. Descriptive statistics were used to summarize the demographic and clinical characteristics of the two groups. Continuous variables are expressed as means and standard deviations, while categorical variables are presented as frequencies and percentages.

For inter-group comparisons, normality of continuous variables was assessed using the Shapiro–Wilk test. Variables satisfying normality assumptions were analyzed using Student’s *t*-test, while non-normally distributed variables were analyzed using the Mann–Whitney U test. Ordinal variables were also analyzed using the Mann–Whitney U test. Nominal variables were compared using the chi-square test or Fisher’s exact test, as appropriate. Statistical tests were selected based on distributional characteristics, and reanalysis was performed where necessary to ensure appropriate test application.

For intra-group comparisons, preoperative and postoperative data were analyzed using paired *t*-tests or the Wilcoxon signed-rank test. A significance level was set at *p* < 0.05.

To minimize selection bias and ensure comparability between the AI-6 and FT-4 groups, propensity score matching (PSM) was employed. The PSM method matched patients based on relevant baseline characteristics, including sex, age, BMI, laterality, concomitant anterolateral ligament (ALL) reconstruction, meniscal lesion, and preoperative anterior laxity (SSD).

The propensity scores were calculated using the PSM process embedded in SPSS, estimating the likelihood of receiving either the AI-6 or FT-4 technique based on these covariates. Patients were then matched 1:1 using the nearest neighbor matching algorithm with a caliper set to 0.1 to maintain balance across the variables in both groups. After matching, differences in clinical outcomes, such as knee stability and functional knee scores, were analyzed between the two groups.

Additionally, standardized mean differences (SMDs) were calculated to assess the balance between groups for variables used in PSM.

## 3. Results

**Figure 3:**
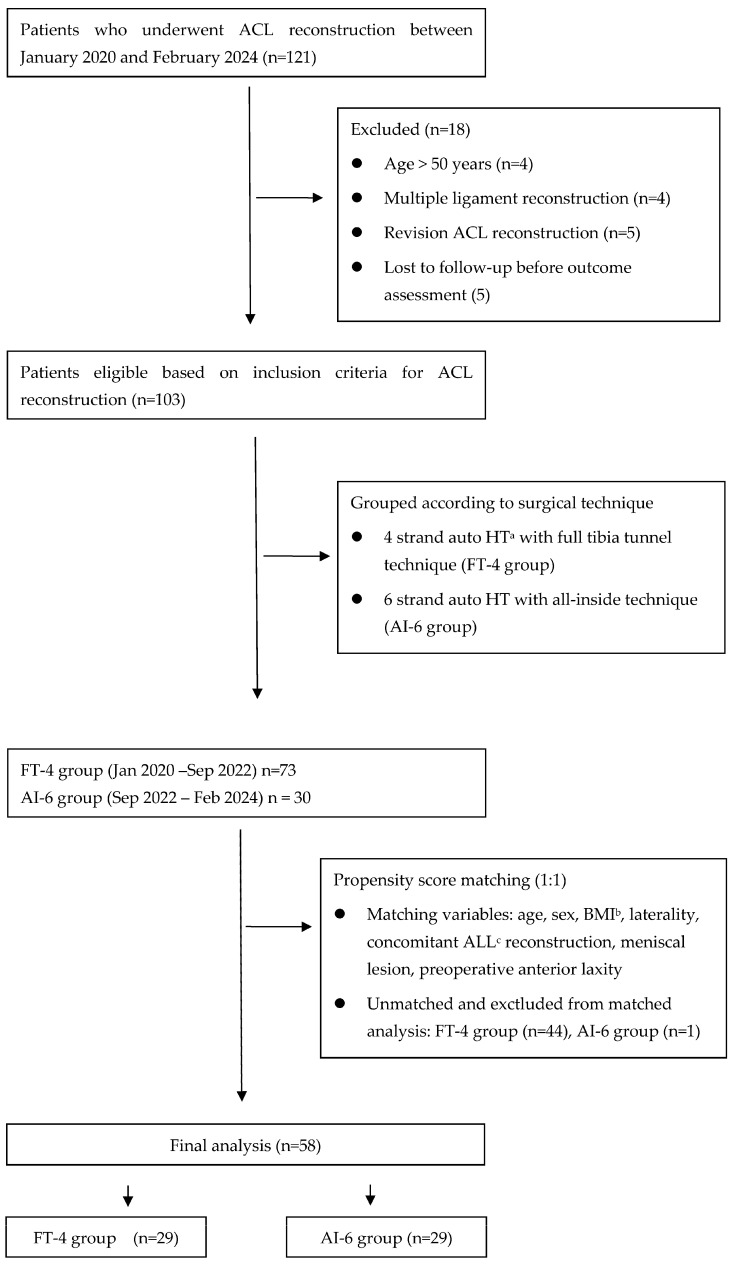
Flow diagram of patient selection and propensity score matching. ^a^ hamstring tendon, ^b^ body mass index, ^c^ anterolateral ligament.

A total of 121 cases were retrospectively reviewed, and 18 were excluded according to the predefined criteria. The remaining 103 patients were included in the analysis, comprising 30 patients in the AI-6 group and 73 in the FT-4 group. After PSM, 29 matched cases were selected for each group (Figure 3).

The mean follow-up duration was 27.1 ± 3.8 months (range, 20.3–35.1 months) in the AI-6 group and 29.6 ± 13.8 months (range, 12.1–57.3 months) in the FT-4 group. Preoperative characteristics of the matched cohorts are summarized in Table 1. Among the variables, graft diameter was significantly greater in the AI-6 group, whereas no significant differences were observed in other baseline characteristics. However, some SMDs exceeded the ideal cutoff of 0.1. Although an SMD below 0.1 is preferred, values up to 0.25 are sometimes considered acceptable. A few variables surpassed this threshold, likely due to small sample size and retrospective design, which should be acknowledged as an important limitation with the potential to affect the validity of the group comparisons.

### 3.1. Within-Group Analysis of Clinical Outcomes

At the final follow-up, analysis of clinical outcomes within each group revealed significant postoperative improvements. In the AI-6 group, postoperative anterior laxity (SSD), IKDC score, and Lysholm score improved to 1.58 ± 1.1 mm, 78.03 ± 7.0, and 82.2 ± 6.7, respectively (all *p* < 0.001). Similarly, the FT-4 group showed corresponding values of 2.5 ± 1.4 mm, 77.2 ± 8.4, and 75.6 ± 8.9 (all *p* < 0.001). Both groups showed significant gains in activity level, with Tegner scores increasing from a median [IQR] of 6 [1.5] in the AI-6 group and 5 [1.5] in the FT-4 group (all *p* < 0.001).

Postoperative pivot shift grades also improved significantly in both groups (all *p* < 0.001), with the AI-6 group showing 16 patients at grade 0, 12 at grade 1, and 1 at grade 2, while the FT-4 group had 16 at grade 0, 9 at grade 1, and 4 at grade 2.

### 3.2. Between-Group Analysis of Clinical Outcomes

Inter-group comparisons of functional knee scores at the final follow-up demonstrated significantly superior outcomes in the AI-6 group for Lysholm and WOMAC scores, whereas no significant differences were observed in IKDC, Tegner, or KKS. A summary of the detailed data is shown in Table 2.

At the final outpatient follow-up, postoperative instability was compared between the two groups, revealing that the AI-6 group demonstrated significantly better results in anterior laxity (SSD), whereas no significant difference was observed between the groups in the pivot-shift test, as detailed in Table 3. The pivot-shift test was conducted in the outpatient clinic by a senior surgeon with extensive experience, ensuring adequate muscle relaxation to reduce the influence of voluntary guarding.

Given that key preoperative variables were balanced through propensity score matching, a simple linear regression was performed to assess the independent association between graft diameter and postoperative anterior laxity. The analysis revealed a significant negative relationship (B = −0.398, β = −0.287, *p* = 0.048; 95% CI: −0.791 to −0.005). Residual analysis showed no major violations of linearity, normality, or homoscedasticity. The residual sum of squares was 100.995 (df = 46) with a mean square error of 2.196. The model showed a modest fit (R = 0.287, R^2^ = 0.083), indicating that a larger graft diameter was associated with reduced postoperative anterior laxity.

A post hoc power analysis was performed using G*Power 3.1 (Heinrich-Heine-University Düsseldorf, Germany), with postoperative anterior laxity selected as the main outcome variable for this analysis due to its clinical relevance. Based on the observed group difference (1.6 ± 1.1 mm in the AI-6 group vs. 2.5 ± 1.4 mm in the FT-4 group), the analysis yielded a Cohen’s d of 0.71 and an estimated statistical power of 85.2% (one-tailed, α = 0.05), indicating that the sample size of 29 patients per group was sufficient to detect a clinically meaningful difference with adequate statistical rigor.

## 4. Discussion

The most important findings of this study were that the six-strand configuration used in the all-inside technique yielded significantly thicker grafts, which resulted in reduced postoperative anterior knee laxity compared to the full tibial tunnel technique. Although some functional outcomes, such as the IKDC, Tegner, and KKS, showed no significant differences between groups, the all-inside group demonstrated superior results in the Lysholm and WOMAC scores. These findings lend partial support to our hypothesis, given that the AI-6 group showed superior anterior knee stability and outperformed the FT-4 group in selected functional assessments.

Previous studies have yielded inconsistent findings regarding the relationship between graft diameter and postoperative knee outcomes, particularly in the context of autograft hamstring reconstruction. Although a direct comparison is limited due to differences in graft configurations, the findings of the current study align with the general trend reported in a recent network meta-analysis, which demonstrated that graft constructs with a greater number of strands, such as four-strands graft with augmentation, were associated with reduced anterior knee laxity compared to lower-strand configurations [18]. These findings suggest that the improved outcomes in the AI-6 group may be partially explained by the increased graft diameter inherent to the six-strand technique, which has been reported as a contributing factor to enhanced postoperative stability [18]. In contrast, a recent meta-analysis of Level I–II studies comparing five-strand and four-strand hamstring autografts demonstrated significantly larger graft diameters in the five-strand group (mean difference of 0.93 mm; 8.5–9.1 mm vs. 7.5–8.1 mm) and found no significant differences in anterior laxity based on the Lachman test [19]. Conversely, our study employing six-strand hamstring autografts revealed significant differences in instrumented anterior laxity. This discrepancy may be explained by both differences in anterior laxity evaluation methods and the distinct graft configurations utilized. Furthermore, this meta-analysis demonstrated that, although some PROMs, such as KOOS ADL and Lysholm scores, favored the five-strand group, the pivot shift test outcomes were similar across both groups, consistent with the findings of our study [19]. It is noteworthy that none of the mean differences in the aforementioned PROMs that favored the five-strand group surpassed the minimal clinically important difference (MCID) thresholds, suggesting comparable overall clinical outcomes [19]. Interestingly, one of the studies included in the aforementioned meta-analysis, by Wan KM et al., reported transitioning to the all-inside technique to accommodate shorter graft lengths, a method similar to the one employed in the present study [16]. They also described that a substantial proportion of Asian patients fail to achieve a graft diameter of 8.5 mm using a conventional four-strand hamstring technique [20,21,22,23].

Consequently, the five-strand technique has been considered particularly beneficial for Asian populations, where smaller graft diameters are more commonly encountered, and can be effectively applied in all-inside ACL reconstruction procedures.

In this context, the all-inside technique has garnered attention as a potential alternative to the traditional full tibia technique. Several recent meta-analyses comparing the two techniques have demonstrated comparable outcomes in terms of postoperative knee function, graft failure, and overall stability [3], while the all-inside technique has shown superior results regarding reduced postoperative pain, improved flexion strength, larger graft diameters, and decreased tunnel widening [1,2]. Upon closer examination focusing specifically on anterior laxity, however, a recent meta-analysis by Zhu et al. reported greater anterior laxity in the all-inside group compared to the full tibia group, which, somewhat surprisingly, contrasts with the findings of our study [24]. Although Zhu et al. speculated that factors such as adjustable loop elongation and the earlier initiation of rehabilitation protocols might contribute to this difference, the true cause remains uncertain. Notably, a detailed review of the three randomized controlled trials included in their analysis revealed that all three studies consistently utilized only the semitendinosus tendon in the all-inside group and both the gracilis and semitendinosus tendons in the full tibia group, resulting in similar graft diameters of approximately 8 mm without statistically significant differences [5,7,8]. Given that our study used a significantly thicker graft in the all-inside group, it is plausible that graft diameter, rather than the surgical technique alone, plays a key role in the differences observed in postoperative anterior laxity. However, further studies are warranted to confirm this notion.

Furthermore, upon closer inspection, two of these studies were conducted at the same institution and merely reported outcomes at different follow-up periods, and did not demonstrate statistically significant differences in anterior laxity [5,8]. Only one study reported a statistically significant difference in anterior laxity between the groups. However, despite the statistical significance, the mean SSD was 2.9 mm in the all-inside group and 2.3 mm in the full tibial tunnel group, which raises questions about the clinical relevance of this difference [7]. This critical discrepancy in graft configuration, sample size, and outcome reporting may account for the opposing findings, as our study demonstrated superior postoperative anterior laxity in the all-inside group, likely attributable to the larger graft diameters achieved in our cohort.

As previously discussed, most studies on the all-inside technique have reported results using only the semitendinosus to construct the graft, a method associated with several advantages, such as reduced soft tissue damage and improved flexor power, owing to gracilis sparing. Given these advantages, the all-inside approach also offers a notable technical merit in its ability to accommodate a graft that is relatively thick despite its short length. Although harvesting the semitendinosus alone may suffice in certain cases, it does not always yield a sufficiently thick graft, particularly when a more robust construct is desired. In our study, combining the semitendinosus and gracilis tendons to create a six-strand graft within the all-inside technique provided a practical and effective solution to achieve adequate graft diameter [10,19,25,26].

A key strength of this study is the direct comparison of two distinct graft constructions using propensity score-matched cohorts, providing practical insight into graft preparation strategies. This highlights the clinical relevance of the six-strand hamstring graft configuration, which enables the use of thicker grafts associated with improved postoperative anterior knee stability and select functional outcomes. Given the higher prevalence of smaller graft diameters among Asian populations, this approach may offer particular value in such cohorts by facilitating adequate graft thickness through multifold techniques. Additionally, the all-inside technique offers a practical solution to accommodate the shorter length inherent to six-strand grafts. However, this approach may introduce technical challenges during graft passage, as both femoral and tibial tunnel insertions must be accessed sequentially through the same portal.

Nevertheless, the purported advantages of the all-inside technique reported in this study should be interpreted with caution, considering the following limitations. First, graft failure was not included as a clinical endpoint in the analysis. Given that several previous studies have indicated a potential association between increased graft diameter and reduced failure rates, omission of this outcome may have limited the comprehensiveness of the findings [10,11,12,13]. Eggeling et al. reported that clinical failure following ACL reconstruction can be defined as postoperative anterior laxity (SSD) greater than 6 mm [27]. From this perspective, although graft failure was not included in the primary outcome analysis of the present study, one patient in the FT-4 group demonstrated postoperative anterior laxity (SSD) exceeding this threshold, thereby meeting the criteria for clinical failure. In contrast, no such cases were observed in the AI-6 group. While this finding was not statistically analyzed due to the limited sample size, it may suggest a potential difference in failure risk between graft configurations, warranting further investigation in future studies with larger cohorts and longer-term follow-up.

Second, when interpreted in light of the MCID, the clinical relevance of the observed inter-group differences warrants careful consideration. Although the AI-6 group showed statistically superior outcomes, the clinical magnitude of these differences varied across outcome measures. Specifically, the Lysholm score showed a mean difference of 6.6 points, exceeding the MCID range reported in prior studies (typically 4–10 points) with a corresponding effect size (Cohen’s d) of 0.85, indicating a large effect [28]. In contrast, the WOMAC score demonstrated a mean difference of 4.9 points, which did not reach the commonly cited MCID threshold of 6–12 points, with an effect size of 0.58, corresponding to a medium effect [29,30]. Regarding anterior laxity, the SSD was 0.9 mm, with an effect size of 0.72. Although statistically significant, this difference may not be clinically perceptible, as MCID values for anterior translation are generally considered to be ≥2 mm [31]. These findings highlight the importance of considering both statistical and clinical relevance when interpreting postoperative outcomes.

Third, while the sample size in this study remains relatively small, the use of propensity score matching helps mitigate potential confounding factors and strengthens the validity of our findings. By matching participants on key baseline characteristics, this approach enhances the comparability between the AI-6 and FT-4 groups, providing a more reliable estimate of the treatment effects despite the limited sample size. Additionally, given the retrospective nature of the study, a formal primary endpoint was not prospectively designated. However, a post hoc power analysis was performed based on the observed difference in postoperative anterior laxity, which yielded a statistical power of 85.2%, supporting the reliability of this outcome measure in our analysis.

Fourth, this study’s retrospective design inherently carries risks of selection bias and potential inconsistencies in data collection. Residual confounding from unmeasured variables cannot be entirely excluded. Additionally, the relatively short-term follow-up limits the assessment of long-term outcomes and graft durability. Therefore, further prospective studies with extended follow-up are warranted to validate and expand upon these findings.

## 5. Conclusions

The present study demonstrated that the use of a six-strand hamstring graft configuration in the AI-6 technique resulted in significantly thicker grafts and was associated with reduced postoperative anterior knee laxity compared to the FT-4 technique. While interpretation of these findings requires caution in light of MCID thresholds, the AI-6 group showed favorable outcomes in anterior laxity and selected functional scores, such as the Lysholm and WOMAC. This technique may offer practical clinical value, particularly in populations prone to smaller graft diameters, as it facilitates adequate graft thickness through multifold preparation, with the all-inside approach accommodating the inherent graft shortening.

## Figures and Tables

**Table 1:** Comparison of patient characteristics after PSM.

Variables ^a^	AI-6 (n = 29)	FT-4 (n = 29)	*p*-Value	SMD ^h^
Age (year) *	27.7 ± 8.3	30.3 ± 12.2	0.629	0.249
Sex *				
Male (%)/Female (%)	18 (62.1)/11 (37.9)	19 (65.5)/10 (34.5)	1.0	0.072
Body mass index (kg/m^2^) *	26.3 ± 3.5	26.5 ± 3.3	0.879	0.040
Side *				
Right (%)/Left (%)	18 (62.1)/11 (37.9)	21 (72.4)/8 (27.6)	0.401	0.221
Time from Injury to surgery (weeks)	3.9 ± 4.5	5.2 ± 5.0	0.249	
Time from surgery to final follow-up (months)	27.1 ± 3.8	29.6 ± 13.8	0.067	
Preop ^b^ anterior laxity (SSD ^c^, mm) *	6.0 ± 1.3	6.0 ± 1.6	0.972	0.009
Preop pivot shift grade under anesthesia (0/1/2/3) (%)	3 (10.3)/13 (44.8)/9 (31.0)/4 (13.8)	0 (0.0)/12 (41.4)/14 (48.3)/3 (10.3)	0.307	
Preop IKDC ^d^	52.6 ± 5.1	49.7 ± 6.5	0.067	
Preop Lysholm	40.6 ± 8.7	43.8 ± 8.2	0.191	
Preop Tenger ^e^	2 (2–3)	2 (2–3)	0.689	
Graft diameter (mm)	9.5 ± 0.7	7.8 ± 0.8	<0.001	
Femoral tunnel creation method (OI/TP) ^f^ (%)	20 (69.0)/9 (31.0)	15 (51.7)/14 (48.3)	0.283	
ALL reconstruction (%) *	20 (69.0)	23 (79.3)	0.368	0.237
Meniscus lesion *				
medial	15	13	0.793	0.138
Repair				
Ramp lesion	9	6		
Midbody-to Ph ^g^ longitudinal tear	4	2		
Ph radial tear	2	3		
Bucket handle tear		1		
Ph horizontal tear		1		
lateral	9	13	0.417	0.287
Repair				
Ph radial tear near root	6	4		
Ph radial tear		4		
Ph longitudinal tear	3	5		

^a^ data are presented as the mean ± standard deviation or the number of patients. Depending on the characteristics of the variables, statistical analyses were conducted using Student’s *t*-test, Mann–Whitney U test, Pearson chi-square test, or Fisher’s exact test. ^b^ preoperative; ^c^ side-to-side difference; ^d^ International Knee Documentation Committee; ^e^ values are presented as median (range); ^f^ OI: outside-in; TP: transportal; ^g^ posterior horn; ^h^ standardized mean difference. * variables used for propensity score matching.

**Table 2:** Inter-group comparison of functional scores.

Variables ^a^	Group	Mean ± SD	*p*-Value
Lysholm	AI-6	82.2 ± 6.7	0.003
	FT-4	75.6 ± 8.9	
IKDC ^b^	AI-6	78.0 ± 7.0	0.684
	FT-4	77.2 ± 8.4	
Tegner ^c^	AI-6	6 [1.5]	0.289
	FT-4	5 [1.5]	
WOMAC ^d^	AI-6	8.0 ± 4.3	0.023
	FT-4	12.9 ± 10.5	
KKS ^e^	AI-6	83.5 ± 7.1	0.528
	FT-4	82.5 ± 7.2	

^a^ data are presented as the mean ± standard; ^b^ International Knee Documentation Committee; ^c^ Values are presented as median [interquatile range]; ^d^ Western Ontario and McMaster Universities Osteoarthritis Index; ^e^ Korean knee score.

**Table 3:** Inter-group comparison of postoperative instability.

Variables ^a^	AI-6	FT-4	*p*-Value
Anterior laxity (SSD ^b^, mm)	1.6 ± 1.1	2.5 ± 1.4	0.005
pivot shift grade (0/1/2/3) (%)	16 (55.2)/12 (41.4)/1 (3.4)/0 (0.0)	16 (55.2)/9 (31.0)/4 (13.8)/0 (0.0)	0.732

^a^ data are presented as the mean ± standard deviation or the number of patients. ^b^ side-to-side difference.

## Data Availability

The original contributions presented in this study are included in the article. Further inquiries can be directed to the corresponding author.
